# Scientific Items

**Published:** 1880-02-20

**Authors:** 


### SCIENTIFIC ITEMS.

**Galvanic Oxidation of Gold.**—New discoveries in chemistry are so common that we cease to wonder at them; and when chemical affinity and electricity work together it is hardly possible to set limits to their power. It has been a familiar statement in text-books of chemistry and technical manuals that gold can be dissolved only by *aqua regia*, or nitro-muriatic acid; but it has recently been discovered that the same result may be attained by the use of dilute sulphuric acid with the aid of the galvanic battery. In a communication to the French Academy of Sciences, M. Berthelot states that his attention was called by M. Chevreul to a remark by Grotthuss, that in his experiments on the decomposition of water by galvanic action he found that the gold wire used as the positive pole in the sulphuric acid through which the current passed was dissolved. M. Chevreul suggested that the action might be due to the formation of persulphuric acid, and M. Berthelot undertook a series of experiments to elucidate the subject. He first of all repeated the experiments of Grotthuss, which he found perfectly correct. Sulphuric acid diluted to one-tenth rapidly dissolved the gold, a portion of which was precipitated on the negative pole. Nitric acid under similar conditions produces the same effect with a violet-colored precipitate (gold or oxide of gold), which remains in suspension. Diluted phosphoric acid, on the contrary, does not affect the gold in any appreciable manner, even under the influences of the galvanic current; nor has potassium any action. The solvent power of sulphuric acid is not due to the presence of ozone; for oxygen charged with ozone does not attack the metal in presence of water, either pure or mixed with sulphuric or nitric acids. Persulphuric acid prepared by electrolysis does not act on gold even when it contains in addition a certain quantity of oxygenated water. The result of M. Berthelot's observation is that the gold is attacked solely under the influence of the galvanic current, and by the contact of the pole with the electrolized liquid.—*Boston Journal of Chemistry.*

**Carrier Pigeons at Great Altitudes.**—Experiments were recently made in Switzerland to ascertain whether carrier pigeons would start at great altitudes, and would find their way from summits covered with snow as well as from less heights. Two pigeons were set at liberty on the Bergli, at a height of 8,600 ft. After perching for a few minutes on a neighboring rock, they took flight in the direction of the Eiger; but soon after they returned to the hut whence they had been liberated. They did not start again for some time, when they took the route for their cot, although, surrounded by mountains, they had not seen the country. Of these two, one did not reach its destination till seven days after; the other failed to appear. Neither (it should be said) had been accustomed to be set at liberty at a great distance from its cot. Another experiment consisted in letting off two pigeons (one of which had not been trained for great distances) about 9:30 a. m., at a point 50 ft. under the highest point of the Jungfrau, or 13,750

ft. above sea-level. They immediately rose, described several large circles, and took their flight down the valley of Lauterbrunnen, in the direction of Schilthorn and Schwalveren. One of these pigeons reached its cot at Thun at three o'clock next day (eight hours after starting). The other did not turn up. The result of these observations is the more interesting, because, in several instances, pigeons let off from balloons high up in the air have seemed to be incapable of sustaining themselves, and have fallen to earth like an inert mass.—*Boston Journal of Chemistry.*.

**Natural Enemies of the Telegraph.**—There is, apparently, no apparatus so liable to be interfered with by what we may call natural causes as the electric telegraph. Fish gnaw and mollusks overweight the submarine conductors of the subterranean wires; while there is at least one instance of a frolicsome whale entangling himself in a deep sea cable, to its utter disorganization. It is stated that within the three years ending 1878, there have been sixty serious interruptions to telegraphic communication, in Summatra, by elephants. In one instance, these sagacious animals most likely fearing snares, destroyed a considerable portion of the line, hiding away the wires and insulators in a canebreak. Monkeys of all tribes and sizes, too, in that favored island, use the poles and wires as gymnasia, occasionally breaking them and carrying off ths insulators; while the numerous tigers, bears and buffaloes on the track render the watching and repair of the line a duty of great danger. In Australia, where there are no wild animals to injure the wires, which are carried great distances over land, they are said to be frequently cut down by the scarcely less wild aborigines, who manufacture from them rings, armlets and other varieties of barbaric ornament. It has been suggested as a means of protection in this case that the posts should be constructed of iron, when the battery could be used to astonish any native climbing them with felonious intent.—*Scientific American.*

**The Colony of Victoria.**—The government of the colony of Victoria has caused to be compiled and published a full account of the aboriginal people of Australia, with notes concerning their customs. This work shows clearly that the Australians are really comparatively a new race to the continent on which they are already fast approaching extinction. Innumerable stone hatchets and basaltic chips, close to the surface of the soil all over the country, attest the former presence of the aborigines everywhtre; but these relics occur only near the surface. No fossil remains of man have yet been met with, even in the alluvial deposits where the gold miners have worked. We have no evidence that man was contemporary with the great fossil kangaroo or the native lion; and, although, says Mr. R. Brough Smyth, the compiler of the present work, the bones of the native dog are found beneath the volcanic ash beds, there is nothing to show that this dog ever had a master. From these and other facts is drawn the inference that the Australian aborigines are a new race to their own land.

A CERMAN chemist is said to have invented an inflammable substance capable of serving as a substitute for white phosphorus in the manufacture of matches.

